# Impact of Diets on Response to Immune Checkpoint Inhibitors (ICIs) Therapy against Tumors

**DOI:** 10.3390/life12030409

**Published:** 2022-03-11

**Authors:** Xin Zhang, Huiqin Li, Xiupeng Lv, Li Hu, Wen Li, Meiting Zi, Yonghan He

**Affiliations:** 1 Department of Clinical Nutrition, The First Affiliated Hospital of Dalian Medical University, Dalian 116011, China; zhang_xin_85@126.com; 2 State Key Laboratory of Genetic Resources and Evolution, Kunming Institute of Zoology, Chinese Academy of Sciences, Kunming 650201, China; lihuiqin@mail.kiz.ac.cn (H.L.); nyhulixx@126.com (L.H.); zimeiting@mail.kiz.ac.cn (M.Z.); 3 Key Laboratory of Healthy Aging Research of Yunnan Province, Kunming Institute of Zoology, Chinese Academy of Sciences, Kunming 650201, China; 4 Department of Oncology, The First Affiliated Hospital of Dalian Medical University, Dalian 116011, China; lvxiupeng@foxmail.com; 5 Department of Geriatrics, The Second Affiliated Hospital of Hainan Medical University, Haikou 570216, China; 6 Department of Endocrinology, The Third People’s Hospital of Yunnan Province, Kunming 650011, China; ssynfm@163.com

**Keywords:** immune checkpoint inhibitor, resistance, diet, tumor

## Abstract

Immunotherapy has revolutionized the established therapeutics against tumors. As the major immunotherapy approach, immune checkpoint inhibitors (ICIs) achieved remarkable success in the treatment of malignancies. However, the clinical gains are far from universal and durable, because of the primary and secondary resistance of tumors to the therapy, or side effects induced by ICIs. There is an urgent need to find safe combinatorial strategies that enhance the response of ICIs for tumor treatment. Diets have an excellent safety profile and have been shown to play pleiotropic roles in tumor prevention, growth, invasion, and metastasis. Accumulating evidence suggests that dietary regimens bolster not only the tolerability but also the efficacy of tumor immunotherapy. In this review, we discussed the mechanisms by which tumor cells evade immune surveillance, focusing on describing the intrinsic and extrinsic mechanisms of resistance to ICIs. We also summarized the impacts of different diets and/or nutrients on the response to ICIs therapy. Combinatory treatments of ICIs therapy with optimized diet regimens own great potential to enhance the efficacy and durable response of ICIs against tumors, which should be routinely considered in clinical settings.

## 1. Introduction

The fields of immunology and oncology have been linked since the end of the 19th century when the surgeon William Coley [1,2] reported that an injection of attenuated bacteria into the sites of sarcoma led to tumor shrinkage. From then on, there have been exponential advances regarding the interaction between immune surveillance and tumor growth and development, and the concept of utilizing the immune system against malignancies, that is, the theory of tumor immunotherapy. Due to the outstanding success in halting even advanced tumors and in prolonging survival in patients with highly aggressive tumors such as melanoma and lung tumors [3,4,5], immunotherapy has recently emerged as a promising treatment option for many patients with tumors, and revolutionized the established therapeutic approach against tumors. It aims to activate the patient’s immune system to kill tumor cells, generally including chimeric antigen receptor (CAR)-T and -NK cell therapy, immune checkpoint inhibitors (ICIs), cytokine therapy, oncolytic viruses, and tumor vaccines [6]. Considering the remarkable success of ICIs in the treatment of selected malignancies and the prominent and long-lasting responses in tumors with high mutational and neoantigen burdens [7], ICIs have been regarded as the breakers of the tumor treatment dilemma.

ICIs are the blocking antibodies against inhibitory immune checkpoints (ICs) targeting the specific mechanisms that tumor cells employ to evade immune system detection [8]. Despite the promising results of ICIs therapy in melanoma [9,10], urothelial carcinoma [11], lung tumors [12,13], colorectal tumors [14], and head and neck squamous cell carcinoma [15], clinical gains are far from universal. In some of the major tumor types (e.g., breast and pancreas), the clinical efficacy of ICIs remains very limited [16,17]. Besides, only a minority of patients exhibit durable responses [18,19]. In view of the lack of universality and durability, there is an urgent need to find safe combinatorial strategies that enhance the response of ICIs for tumor treatment.

Diet accounts for approximately 30% of the attributable risk for tumors [20]. It was well documented that diets and/or nutrients exerted pleiotropic effects on tumor growth, invasion, and metastasis [21,22,23]; thus, diet has been proposed as an integral part of anti-tumor regimens. Moreover, some nutrients have been shown to play an important role in modulating immune functions [21,24,25]. Thus, dietary regimens have been applied to bolster not only the tolerability but also the efficacy of tumor immunotherapy [25,26]. In this review, we systematically summarized the impacts of diet and/or nutrients on the response of ICIs for tumors.

## 2. Immune Evasion and ICIs

Normally, tumor-associated antigens (TAA) expressed by tumors can be recognized by the immune system, and tumor cells are then eradicated by the tumor-infiltrating lymphocytes (TILs) [27]. However, tumors can create a sanctuary against the immune system and evade immune surveillance. Established mechanisms include:

(1) Loss or alteration of specific antigens or antigenic machinery [28,29]. Tumors can lose major histocompatibility complex (MHC) class I expression or the intracellular machinery required to transport tumor antigens to the tumor surface for T cell recognition [30,31,32].
(2) Tumors can promote an immune-tolerant microenvironment by manipulation of cytokines (increased secretion of IL-6, IL-10, and TGF-beta; consumption of IL-2) that encourage infiltration of regulatory T (Treg) cells, myeloid-derived suppressor cells (MDSCs), and other cell types that inhibit cytotoxic T cell function [31,33]. These cells can actively suppress the proliferation of CD4^+^ and CD8^+^ T lymphocytes that would otherwise recognize tumor antigens.
(3) Tumors can upregulate the expression of inhibitory ICs such as programmed cell death-ligand 1 (PD-L1), which binds to programmed cell death protein 1 (PD-l) on T cells and, thus, promotes peripheral T cell exhaustion [34,35].
(4) Tumors can release acidic and toxic metabolites or deplete nutrients and oxygen to inhibit the activity of immune cells in the tumor microenvironment (TME) [36,37].
(5) Cancer-associated fibroblasts (CAFs) create an immunosuppressive TME. CAFs are the major components of the tumor stroma; they secrete cytokines, chemokines, inhibitory and extracellular matrix (ECM) remodeling molecules that suppress NK cell and cytotoxic T cell activity [38].

**Figure 1:**
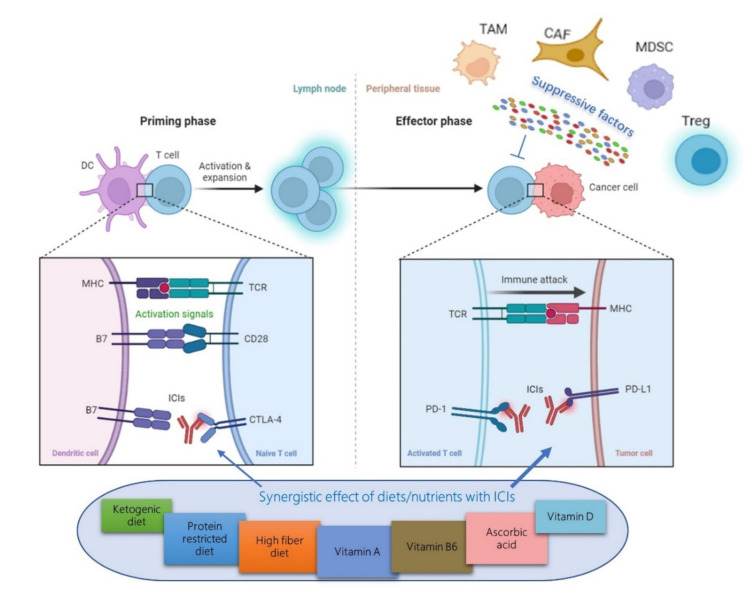
Immune evasion by tumors and effects of dietary factors on immune checkpoint inhibitors (ICIs) therapy. In the priming phase, T cells are activated by antigen-presenting cells (APCs) when T cell receptor (TCR) binds to the antigen displayed in the major histocompatibility complex (MHC) on APCs, e.g., dendritic cells (DCs), in concert with CD28:B7-mediated co-stimulation. In the case of a strong TCR stimulus, CTLA-4 expression is upregulated and competes with CD28 for binding B7 molecules. High levels of CTLA-4:B7 binding limit the survival of T cells and protect tumor cells from T cell attack. In the effector phase, prolonged tumor antigen stimuli cause an upregulation of PD-1 which binds to PD-L1 expressed by tumor cells. PD-1:PD-L1 binding leads to the exhaustion of T cells, which results in immune evasion by tumors. In addition, there are several types of cells, including regulatory T cells (Tregs), myeloid-derived suppressor cells (MDSCs), tumor-associated macrophage (TAMs), and cancer-associated fibroblast (CAFs), which can inhibit the anti-tumor T-cell response via secreting various molecules. Immune checkpoint inhibitors (ICIs), e.g., anti-CTLA-4 and PD-1/PD-L1 antibodies, can reactivate the immune response of T cells to tumors. Emerging evidence suggests that certain dietary patterns and vitamins can synergistically enhance the efficacy of ICIs therapy by affecting the expression of immune checkpoint proteins.

IC molecules can be divided into two categories, stimulatory and inhibitory ICs. Normally, inhibitory ICs act as the natural brakes of the human body via suppressing the body’s immune response and preventing the occurrence of autoimmunity [39]. However, tumor cells can exploit this mechanism to escape the immune system by upregulating the expression of suppressive molecules that interact with T cells, rendering them incapable of killing [27]. For example, the binding of PD-L1 expressed by tumor cells to PD-1 on the surface of TILs weakens the immune role of T cells, which causes tumor immune escape and promotes tumor progression [40]. To date, more than ten types of ICs have been discovered, such as PD-1, PD-L1, cytotoxic T lymphocyte-associated antigen-4 (CTLA-4), T cell immunoglobulin and mucin domain containing-3 (TIM-3), and lymphocyte-activation gene 3 (LAG3/CD223), of which the most extensively studied are CTLA-4 and PD-1/PD-L1. ICIs are the blocking antibodies against inhibitory ICs, which can bind to and inhibit the activity of inhibitory ICs and reactivate the immune response of T cells to tumors (Figure 1).

## 3. Resistance to ICIs

ICIs therapy has become one of the most successful anti-tumor strategies to date. As the representative of ICIs therapy, PD-1/PD-L1 or CTLA-4-based IC blockade therapy has been approved for treating multiple tumor types; however, the efficacy varies in different tumor types [41,42]. Some patients never respond to the treatment (termed as innate resistance), while the responders often develop resistance (termed as acquired resistance). ICIs resistance can be classified as intrinsic or extrinsic according to the sites where resistance happens. For intrinsic resistance, tumor cells acquire resistance to ICIs via altering processes associated with DNA damage response, cell signaling pathways or immune recognition. The exact mechanisms include: (1) tumor neoantigen presentation, structure, and processing are genetically or epigenetically altered, which, thus, influences the activation of immune response and recruitment of effector T cells [7]; (2) alterations in the structure of MHC II/I due to gene defects affect antigen presentation and, ultimately, immune response [38]; (3) dysfunctional interferon (INF) signaling pathway causes inadequate anti-tumor T cell effector function [43,44]. Extrinsic resistance occurs external to tumor cells throughout the T cell activation process, and the influencing factors include TME, host factors such age, genetic background, comorbidities, diet, use of antibiotics and steroids, metabolism, and gut microbiota [18,45,46]. However, to what extent and by what exact mechanisms the extrinsic resistance affects the therapeutic response must be further explored in the future.

## 4. Diet and Response to ICIs

IC inhibitor therapy has brought a paradigm shift in the treatment of advanced tumors by introducing immunotherapy as a recognized first and second-line modality, but much remains to be done to extend its efficacy [18]. Regarding the important roles of nutrients in modulating immune functions and influencing tumor growth and/or responsiveness to immune modulation [45], many dietary regimes are currently being explored in order to enhance the immunotherapy responses in tumor treatments [31]. Although the effects of certain dietary patterns and several nutrients (both macro- and micro-) on immunological outcomes and TME reprogramming have been extensively elucidated [21,23,24,26,47], not all of them were reported to associate with tumor immunotherapy efficacy. Herein, we focus on summarizing the classical effect and data of reported dietary regimens and/or nutrients on the response to ICIs therapy (Table 1).

### 4.1. Ketogenic Diet

The ketogenic diet (KD) was established in the early 20th century, consisting of high fat, low to moderate protein, and very low carbohydrate [71]. The traditional KD is a 4:1 formulation of fat content to carbohydrate plus protein. A classic 4:1 KD delivers 90% of its calories from fat, 8% from protein, and only 2% from carbohydrate. In the 1920s and 1930s, KD was broadly used to treat children with epilepsy [72]. In 1987, Tisdale et al. [73] observed a reduction in tumor weight and improved cachexia in mice with colon adenocarcinoma xenografts consuming a KD. Later, multiple lines of evidence suggest the use of KD as tumor treatment or prevention methods, either alone or in combination with medicines [74,75]. In 2016, the effects of KD on anti-tumor immunity were assessed in a mouse model of glioblastoma [48]. It has been shown to enhance anti-tumor primary and acquired immune responses. Specifically, KD stimulated cytokine production and promoted cytolysis mediated by CD8^+^ T cells, and increased CD4^+^ T cell infiltration as well as T cell killing activity. It may also overcome several immune-escape mechanisms by downregulating the expression of ICs CTLA-4 and PD-1 on TILs as well as the expression of PD-L1 on glioblastoma cells. In parallel, it was observed that KD-like milieus downregulated the expression of cell membrane-associated PD-L1 in an experimental model of highly aggressive basal-like breast tumors [49]. These findings support the notion that KD could impact the expression of ICs on tumor cells and/or that on T cells and, consequently, the responsiveness to ICIs. The composition of the gut microbiota was known to impact the efficacy of ICIs [76,77]. Remarkably, the KD has been shown to increase the relative gut microbiota abundance of *Akkermansia muciniphila* [78], a bacterium capable of ameliorating therapeutic responses to ICIs. Inspiringly, it was recently reported that KD can enhance the efficacy of anti-CTLA-4 immunotherapy by decreasing PD-L1 protein levels in breast tumor cells [50]. Mechanistically, the KD diet activates AMP-activated protein kinase (AMPK), which, in turn, phosphorylates PD-L1 on Ser283, thereby disrupting its interaction with CKLF-like MARVEL transmembrane domain containing 4 (CMTM4) and subsequently triggering PD-L1 degradation. KD or its principal ketone body, 3-hydroxybutyrate (3HB), induced T cell-dependent tumor growth retardation in melanoma models and reestablished the therapeutic responses in conditions under which anti-PD-1 alone or in combination with anti-CTLA-4 failed to reduce tumor growth [51]. Despite that the KD enhanced the response of ICIs in tumor treatment in animal models, clinical data are still very limited. Thus, studies with large cohorts, standardized protocols, and clear indications of compliance are needed and will be awaited.

### 4.2. Protein Restricted Diet

As a key macronutrient and a source of energy, dietary protein plays significant roles in maintaining health. For many years, protein-rich diets have been recommended due to their satiety-inducing and muscle-building effects [79,80,81,82,83]. It was proposed for tumor patients to follow a high protein diet to maintain health during therapy [84]. However, the long-term retrospective and prospective cohort studies have found that a high protein intake was linked to tumor progression and overall mortality [85]. Thus, people hypothesize that limiting protein intake may have benefits in patients with tumors. Indeed, the health benefits of dietary protein restriction for health was first reported in 1928 when McCay et al. [86] found that trout fed a low protein diet lived longer. Recently, it was shown that dietary protein restriction was associated with lowered tumor incidence and mortality risk and inhibited tumor growth in animal models [87]. Protein-restricted diets can be formulated either by reducing the number of amino acids (AAs) and/or reducing the dietary protein intake. Dietary protein restriction (but not carbohydrate) and the resulting decrease in AAs has been shown to induce the activation of endoplasmic reticulum (ER) stress in tumor cells, leading to an anti-tumor T cell response [88]. Of note, AA metabolism plays an important role in determining the fate and function of tumor cells and TILs. It was found that inhibition of arginine synthesis suppressed the proliferation of tumor cells and inhibited the accumulation of Tregs which play an immunosuppressive role [89]. Based on this, the combination of arginase inhibitor and IC therapy is under clinical trials. In addition, CD8^+^ T cells dealt with a specific inhibitor of glutamine metabolism effectively eliminated tumor cells and reduced PD-1 expression [52], indicating that the inhibition of glutamine metabolism prevents the exhaustion of CD8^+^ T cells. Given the indispensable role of anti-tumor T response in ICIs therapy, Orillion et al. [53] tested the effect of a protein-restricted diet on the response to anti-PD-1 immunotherapy in animal models of the prostate (RP-B6Myc) and renal cell carcinoma (RCC). Interestingly, they found that dietary protein restriction strengthened the capacity of TAMs to kill tumor cells and significantly increased the effects of ICIs on tumor growth. Mechanistically, a protein-restricted diet inhibits the mTOR pathway and selectively induces the death of TAMs M2 subtype (immune-suppressive), a shift towards M1 subtype (immune-activating), and impairment of MDSCs development. Later, another study revealed that dietary deprivation of non-essential AAs improved the anti-PD-1 immunotherapy in a mouse model of colon tumors [54]. Though a protein-restricted diet has shown a very promising role in ICIs therapy, there remain many questions. For example, what is the exact amount of protein referred to as a protein-restricted diet? Which AA is the most effective when combined with ICIs therapy? The last, but most important, question is, can the protein-restricted diet be translated into the clinic?

### 4.3. High Fiber Diet

Dietary fibers are edible carbohydrate polymers with 3 to 9 monomeric units resistant to endogenous digestive enzymes and, thus, are neither hydrolyzed nor absorbed in the upper part of the digestive tract. According to their solubility, they are categorized as water-soluble or -insoluble, and, according to their fermentability, they are categorized as nonfermentable, partially fermentable, or completely fermentable [90]. Fiber intake has been ascertained as an essential component of a healthy diet [91]. Reliable associations have been observed between a higher dietary fiber intake and a lower risk of developing neoplasms, including certain gastrointestinal tumors, such as colon and rectal tumors, and colorectal adenoma [92,93,94]. The beneficial roles of dietary fiber may be attributable to their physical, immunomodulatory, and prebiotic activities [95]. Physically, carcinogens can be diluted in a large amount of stool resulting from the ingestion of nonfermentable fiber [96]. Besides, dietary fiber fermentation can reduce fecal pH which further decreases the production of bacterial carcinogens deriving from bile acid metabolism [97]. Bowel intraluminal fiber could be fermented by gut microbiota, forming short-chain fatty acids (SCFAs), predominantly acetate, butyrate, and propionate. SCFAs exhibit immunomodulatory functions in the host by affecting CD4^+^ T cell differentiation, T effector/regulatory T cell balance, and enhancing the generation of antigen-presenting cells [98]. Butyrate has the activity of enhancing CD8^+^ T cell memory function [99]. Therefore, fiber is regarded as an immunomodulatory nutrient. Last, but not least, some types of fibers have a prebiotic effect. Upon being fermented in the colon, they selectively promoted the growth or enhanced the activity of the microbiota [90], maintaining gut homeostasis and increasing microbial richness and diversity. Compelling evidence demonstrated that fibers are indispensable to both spontaneous tumor-specific T cell responses as well as subsequent responses to ICIs [55,56,57,58,77,100,101,102]. Based on these findings, Spencer et al. [59] hypothesized that a high fiber diet may associate with improved responses to ICIs therapy. They found that melanoma patients with a high-fiber diet were five times more likely to respond to anti-PD-1 therapy compared with those with low-fiber diets (OR = 5.3, 95% CI: 1.02–26.3). These findings suggest that a high fiber diet is beneficial to reduce the risk of tumor occurrence and significantly improve the response to ICIs therapy.

### 4.4. Micronutrients

#### 4.4.1. Vitamin D

Vitamin D is a fat-soluble vitamin, including both cholecalciferol and ergocalciferol, which have the common effect of preventing or curing rickets in children and osteomalacia in adults [103,104]. Vitamin D can be viewed as a hormone since it can be synthesized in the skin through the action of ultraviolet rays upon the precursors, 7-dehydrocholesterol and ergosterol, that acts on vitamin D receptors (VDR) to regulate not only calcium homeostasis but also various physiological activities, including immune system modulation [105]. Specifically, the overall effect of vitamin D on the immune system consists of stimulating innate immunity and inhibiting adaptive immunity [104,106]. Dimitrov et al. [60] elucidated that 1,25-dihydroxy vitamin D (1,25-(OH)2-D), the active form of vitamin D, suppressed the activation of CD4^+^ and CD8^+^ T cells and inhibited inflammatory cytokine production in humans by stimulating the gene expression of PD-L1. The finding suggests that elevated vitamin D signaling in humans may suppress anti-tumor immunity. Indeed, the anti-tumor properties of vitamin D have been well elucidated [107,108,109,110] since Colston et al. [111] first described the inhibitory effect of 1,25-dihydroxy vitamin D3 on the proliferation of melanoma cells in 1981. To date, Vitamin D has been shown to exert pleiotropic antineoplastic activities by inhibiting tumor cell growth [112], promoting the differentiation of tumor cells towards a normal or less malignant phenotype [113], suppressing inflammation and angiogenesis [114], and reducing the metastatic potential of tumor cells [115]. More importantly, melanoma patients could potentially benefit from the concomitant administration of vitamin D and ICIs based on the following observations [106]: (1) Vitamin D has an anti-proliferative effect in experimental melanoma models [61], which could strengthen the cytotoxic activity of T cells stimulated by ICIs; (2) Vitamin D was shown to upregulate the expression of PD-L1 [60], which was positively correlated with the response to ICIs in melanoma [116]. The upregulation of PD-L1 by vitamin D in humans may be a double-edged sword for ICIs in tumor immunotherapy. Further clinical investigations are needed to establish an effective PD-L1 cutoff and, as a consequence, an effective vitamin D concentration for ICIs therapy.

#### 4.4.2. Ascorbic Acid

Vitamin C is a water-soluble vitamin that exists in both reduced and oxidized forms [117,118,119]. As the former is present in the plasma at a much higher concentration, it represents the overall level of vitamin C. The reduced form of vitamin C is called ascorbic acid (ascorbate). This name originated from Latin with the meaning of “without scurvy”, a disease caused by vitamin C deficiency characterized by bleeding gums and poor wound healing, which was once common in sailors at sea whose diet was lacking in fresh fruits (especially citrus fruits) and vegetables [119,120]. In the 1930s, vitamin C was discovered not just as a nutritional supplement but also as an anti-microbial agent [121,122]. In the clinic, it is employed for immunity support and reducing therapeutic side effects for tumor patients. In 1978, a clinical trial by Cameron and Pauling showed that intravenously administered high-dose vitamin C had beneficial effects on the survival time of terminal tumor patients [123]. In contrast, two randomized double-blinded clinical trials showed no beneficial effects with high-dose vitamin C therapy [124,125]. The discrepancies were likely due to the different routes of administration (oral or intravenous) [117,126]. Clinical evidence supports that the anti-tumor properties of vitamin C are effective when 1 g/kg dose was administered intravenously over 2 h twice weekly or more frequently [127]. Applied as an adjuvant, a high-dose vitamin C could enhance the effect of chemotherapy [128,129,130], whilst its administration amongst patients with tumors increased the quality of life, improved the physical, mental and emotional conditions, and decreased adverse effects of chemotherapy [131]. In 2019, vitamin C was reported to activate ten-eleven translocation-2 (TET-2), which further regulates the PD-L1 expression by interferon γ (IFN γ)-JAK-STAT signaling pathway and, thus, promoted the immunotherapy [62]. Therefore, vitamin C is expected to promote tumor immune response to ICIs therapy. Inspiringly, several recent studies in murine tumor models have shown that a high dose of vitamin C synergized with ICIs therapy (anti-PD-1 with or without anti-CTLA-4) in several tumor types [63,64]. Specifically, a high-dose of vitamin C increased the infiltration of CD4^+^ and CD8^+^ T cells and macrophages into TME, increased the production of granzyme B by CD8^+^ T cells and interleukin-12 by macrophages, and suppressed tumor growth in a T cell–dependent manner. In addition, vitamin C was shown to markedly improve chemokine and PD-L1 expression that was associated with an increased number of TILs and improved anti-tumor immunity, as well as with enhancing the efficacy of anti-PD-L1 immune therapy [62].

#### 4.4.3. Vitamin A

Vitamin A refers to retinol and derivatives of retinol occurring in two main forms: preformed vitamin A (retinol, retinal, retinoic acid, and retinyl esters) and provitamin A (such as alpha-carotene, beta-carotene, beta-cryptoxanthin, gamma-carotene) [132]. Vitamin A cannot be synthesized in the human body, it must be supplied from the diet [133]. The important role of vitamin A in visual health was already known as early as around 1500–1800 B.C. when the ancient Egyptians recommended compressed animal livers (the richest source of vitamin A) for the treatment of night blindness [134]. Vitamin A is also a regulator of cell differentiation and immune response [135,136]. Retinoids, the derivatives of vitamin A, are the best-studied chemo-preventive agents for various diseases and are used in clinical practice for chemoprevention and treatment of several tumors [137,138]. All-trans retinoic acid (ATRA), an active biological metabolite of vitamin A, has been shown to be a chemotherapeutic agent in the treatment of acute promyelocytic leukemia (APL) [139]. Retinoic acid (RA) therapy has also been shown to improve the survival of neuroblastoma patients [140]. ATRA was also found to inhibit cell growth, induce apoptosis, and downregulate PD-L1 expression in oral squamous cell carcinoma (OSCC) [65], indicating ATRA as a potential alternative adjuvant to ICIs therapy in OSCC. Circulating MDSCs are correlated with decreased responses to immunotherapy in melanoma patients [141,142], and were reported to promote tumor growth by producing immunosuppressive molecules in TME, such as IL-10, and reactive oxygen species (ROS), as well as expressing cell surface receptors, such as PD-L1 [143,144]. Strikingly, Tobin et al. [66] found that the combination of CTLA-4 antibody with ATRA significantly decreased the number of circulating MDSCs with increased activated CD8^+^ T cells. Additionally, ATRA reduced the expression of immunosuppressive genes, including PD-L1, IL-10, and indoleamine 2,3-dioxygenase in MDSCs. On the contrary, ATRA in TME sometimes caused tumor resistance to PD-1/PD-L1 blocking antibodies through upregulation of CD38 [67]. In murine sarcoma models, tumor-derived retinoic acid (RA) blocked monocyte differentiation into dendritic cells (DCs) within the TME to promote immune suppression, whilst blocking RA production in tumor cells or inhibiting RA signaling within the TME increased the percentage of immunostimulatory antigen-presenting cells (APCs), engendered T cell-dependent anti-tumor immunity, and showed strong synergy with anti-PD-1 therapy [68].

#### 4.4.4. Vitamin B6

Vitamin B6 comprises six interconvertible vitamers, including three naturally occurring forms [145], pyridoxine (PN), pyridoxal (PL) and pyridoxamine (PM), and three phosphorylated counterparts [146], pyridoxal 5′-phosphate (PLP), pyridoxine 5′-phosphate (PNP), and pyridoxamine 5′-phosphate (PMP). PLP, the mainly bioactive form of vitamin B6, serves as a co-factor and catalyzes more than 150 biochemical reactions for cellular and organismal metabolism and functions within the endocrine, neurological and immune systems [145,147]. Vitamin B6 is one of the B complex vitamins, which are important contributors of nutritional support to the immune system. Its deficiency can alter immune function considerably by disturbing nucleic acid production and protein synthesis, impeding the maturation and growth of lymphocytes and impairing the production of antibodies and T cells activity [69,148]. Abundant epidemiological studies have validated that dietary vitamin B6 intake was correlated with reduced tumor incidence [149]. Meanwhile, an elevated level of the enzyme, pyridoxal kinase (PDXK), which facilitates the conversion of PN into PLP, was reported as a good prognostic marker in patients with non-small cell lung carcinoma [147]. In 2012, vitamin B6 was found to sensitize a large panel of tumor cells to apoptosis by exacerbating cisplatin-mediated DNA damage [150]. In 2019, Mikkelsen et al. [69] investigated the effect of B vitamins on the modulation of the immune response, they found that vitamin B6 exerted anti-proliferative and anti-migratory activities in promonocytic lymphoma cell lines, likely by decreasing the expression of PD-L1, indicating the potential of vitamin to improve the efficiency of PD-1/PD-L1 blockade. In 2021, Yuan et al. [70] tested and verified that vitamin B6 can act as a PD-L1 suppressor and block the PD-1/PD-L1 signaling pathway, suggesting that an appropriate supplement of vitamin B6 has the potential to increase the efficacy of immunotherapy. These findings may lay the basis for future research into the use of vitamin B6 supplementation to enhance the efficacy of ICIs therapy in vivo.

## 5. Conclusions and Perspectives

ICIs have revolutionized the therapeutic landscape for multiple malignancies. Some patients with untreatable tumors enjoy their lifespan even overpassing the most wishful predictions. However, these outcomes have yet to become the norm due to innate and acquired resistance. Thus, the ultimate challenge is to maximize the efficacy of ICIs therapy. In this review, we summarized the impacts of certain dietary patterns and micronutrients on response to ICIs therapy in tumors. To sum up, the three dietary patterns (KD, protein-restricted diet, and high fiber diet) and the two vitamins (ascorbic acid and vitamin B6) have been demonstrated to enhance the efficacy of ICIs therapy both in vitro and in vivo with causal associations, while the effects of vitamin A are still controversial. Concomitant administration of vitamin D and ICIs could potentially benefit melanoma patients. Combinations with dietary regimes showed great potential to provide adjuvant tools in enhancing the efficacy of ICIs against tumors, which could be introduced by patients themselves under the guidance of professional dieticians. Unfortunately, since parameters correlating dietary habits with clinical outcomes during anti-tumor therapy are not routinely considered, and the current findings are mainly obtained in animal models and/or short-term clinical and observational studies (Table 1), randomized controlled clinical trials are still lacking in this regard. Future studies with detailed information about dietary administration during ICIs therapy trials will be awaited with great interest.

## Figures and Tables

**Table 1:** Effect of diets on the response to ICIs therapy in tumors.

Diet/Nutrients	Impact on the Expression of ICIs and/or Outcome for ICIs Therapy	Source of Evidence	References
Ketogenic diet	Downregulate the expression of CTLA-4, PD-1 on TILs and PD-L1 on glioblastoma	Animal model	[48]
	Downregulate the content of cell membrane-associated PD-L1	Tumor cells	[49]
	Enhance the efficacy of anti-CTLA-4 immunotherapy by decreasing PD-L1 protein levels	Tumor cells	[50]
	Reestablish therapeutic response when anti-PD-1 alone or in combination with anti-CTLA-4 failed to reduce tumor growth	Animal model	[51]
Protein restricted diet	Deprivation of glutamine reduces PD-1 expression	Animal model	[52]
	Increase the effects of ICIs on tumor growth	Animal model	[53]
	Deprivation of non-essential amino acids improves anti-PD-1 immunotherapy	Animal model	[54]
High fiber diet	Increase microbial richness and diversity thus enhance response to ICIs therapy	Clinical study	[55,56,57,58]
	Enhance response of ani-PD-1 therapy	Clinical study	[59]
Vitamin D	Stimulate transcription of the gene encoding PD-L1	Human epithelial and myeloid cells	[60]
	Strengthen the cytotoxic activity of T cells stimulated by ICIs	Tumor cells	[61]
Vitamin C	Improve PD-L1 expression	Tumor cells	[62]
	Synergize with ICIs therapy (anti-PD-1 with or without anti-CTLA-4)	Animal model	[63,64]
Vitamin A	Downregulate PD-L1 expression	Tumor cells	[65]
	Reduce the expression of the gene encoding PD-L1	Clinical study	[66]
	Cause tumor resistance to PD-1/PD-L1 blocking antibodies	Animal model	[67]
	Suppress anti-PD-1 therapy	Animal model	[68]
Vitamin B_6_	Decrease expression of PD-L1	Tumor cells	[69]
	Suppress PD-L1 expression and block the PD-1/PD-L1 signaling pathway	Tumor cells	[70]

## Abbreviations

AAs	amino acids
AMPK	AMP-activated protein kinase
APCs	antigen-presenting cells
ATRA	all-trans retinoic acid
CAFs	cancer-associated fibroblasts
CAR	chimeric antigen receptor
CMTM4	CKLF like MARVEL transmembrane domain containing 4
CTLA-4	cytotoxic T lymphocyte-associated antigen-4
DCs	dendritic cells
ER	endoplasmic reticulum
ICs	immune checkpoints
ICIs	immune checkpoint inhibitors
INF	interferon
KD	ketogenic diet
MDSCs	myeloid-derived suppressor cells
MHC	major histocompatibility complex
PD-l	programmed cell death protein 1
PD-L1	programmed cell death-ligand 1
PL	pyridoxal
PM	pyridoxamine
PMP	pyridoxamine 5′-phosphate
PN	pyridoxine
PNP	pyridoxine 5′-phosphate
RA	retinoic acid
RCC	renal cell carcinoma
SCFAs	short-chain fatty acids
TAAs	tumor-associated antigens
TAMs	tumor-associated macrophages
TILs	tumor-infiltrating lymphocytes
TME	tumor microenvironment
